# The International Guideline Evaluation Screening Tool (IGEST): development and validation

**DOI:** 10.1186/s12874-022-01618-5

**Published:** 2022-05-10

**Authors:** Daniela D’angelo, Daniela Coclite, Antonello Napoletano, Silvia Gianola, Greta Castellini, Roberto Latina, Laura Iacorossi, Alice Josephine Fauci, Primiano Iannone

**Affiliations:** 1 National Center for Clinical Excellence, Healthcare Quality and Safety, Istituto Superiore di Sanità, Via Giano della Bella, 34, 00162 Rome, Italy; 2 Unit of Clinical Epidemiology, IRCCS Istituto Ortopedico Galeazzi, Milan, Italy; 3 PROMISE Department, University of Palermo, Palermo, Italy; 4 Regina Elena National Cancer Institute, Rome, Italy

**Keywords:** Clinical guideline, Assessment, Development, Validity, Tool

## Abstract

**Background:**

Guideline adaptation provides an important alternative to de novo guideline development by making the process more efficient and reducing unnecessary duplication. The quality evaluation of international guidelines is an essential part of the adaptation process.

The study aims at describing the development and validation of a new tool to screen trustworthy Clinical Practice Guidelines (CPGs) for their adoption/adaption: the International Guideline Evaluation Screening Tool (IGEST).

**Methods:**

The process of developing the IGEST involved two main phases: 1) tool development and 2) content validation. The tool development phase comprised three stages, where the scope of the IGEST was defined and the item pool was generated and refined. The content validation was performed through the computation of a content validity index (CVI) based on the opinions of an expert panel.

**Results:**

All the items obtained a CVI >0.78, which resulted in the validation of the instrument. The final instrument comprised four preliminary conditions and 12 criteria organised into three dimensions: (i) the management of conflict of interest; (ii) the quality of evidence and the coherence between evidence and recommendations; and (iii) the panel composition.

**Conclusion:**

The IGEST showed good content validity for assessing the quality of international guidelines. Using the new tool to select trustworthy guidelines might increase the likelihood that international clinical practice guidelines will be adopted/adapted to the local context by allowing a quick screening of existing guidelines trustworthiness and providing an acceptability threshold that supports the decision-making process.

**Supplementary Information:**

The online version contains supplementary material available at 10.1186/s12874-022-01618-5.

## Introduction

Clinical practice guidelines (CPGs) include recommendations intended to assist individuals, populations, and health care services in the decision-making process [1–4]. Currently, numerous CPGs exist on a range of topics, but they continue to have variable quality, and most of them are not evidence-based or methodologically rigorous [4–7]. In Italy, the Law n. 24/2017 confers CPGs a particularly important role for medical liability, tying the issue of safety and quality of care to adherence to CPGs validated by the Italian National Institute of Health (Istituto Superiore di Sanità, ISS) as a methodological guarantor of the national CPGs produced. However, the quality and number of Italian CPGs has been unsatisfactory so far, and only a small number of guidelines produced by Italian scientific societies is currently available in the national guidelines repository system (Sistema Nazionale Linee Guida, SNLG) [8].

As stated by the ISS methodological manual [9] and by international CPG development standards [3, 10, 11], guideline developers can choose among ‘de novo’, adoption, or adapting high-quality existing recommendations to their own context. The latter offers the advantage of saving time, expertise, and resources by building on previous general guidelines while limiting unnecessary duplication and enhancing applicability [10, 12]. Numerous adaptation methodologies have been proposed [10, 13, 14], and although a preliminary key step is the evaluation of the retrieved guidelines, a specific tool to quickly screen and select high-quality and trustworthy guidelines to be adopted/adapted is lacking.

Several tools aimed at quality assessment have been developed, such as the AGREE instruments [15], the Extent of Adherence to Trustworthy Standards (NEATS) [16], the G-I-N tool [17], and the Right statement [18]. However, a specific tool adequately serving the purpose of screening trustworthy CPGs for their adoption/adaption requires different characteristics from those previously developed. For this purpose, its items should be few and broad enough to provide an overall picture of the methodological quality of guidelines and provide an acceptability threshold. In addition, it should have a user-friendly format to allow developers to screen CPGs’ quality by themselves and in a relatively short time. Therefore, with the purpose of responding to these characteristics, the ISS group developed and validated the International Guideline Evaluation Screening Tool (IGEST) to select high - quality and trustworthy guidelines to be adopted/adapted to a national context. The aim of this study is to describe the development and validation of this new tool.

## Methods

**Fig. 1:**
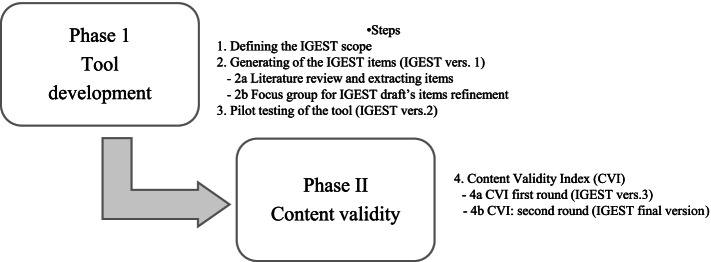
Synthesis of the steps adopted for the development and validation of the IGEST

The process of developing the IGEST involved two main phases, each divided into intermediate steps: phase I - tool development (3 steps), where the scope of the IGEST was defined and the item pool was generated and refined, and phase II - content validity (1 step), where the content validity index (CVI) was established [19, 20]. The whole process is illustrated in Fig. 1.

### Phase 1 - Tool development

#### Define the scope (step 1)

The ISS project steering group with experience in CPG development process defined the scope of the IGEST (step 1, Fig. 1) by specifying the domain and its boundaries [21, 22].

Considering the goal of the IGEST as a tool for screening international trustworthy CPGs for their adoption/adaption in the local context, the attention was directed toward aspects known empirically to most affect the guidelines’ trustworthiness [17, 23, 24], such as a rigorous process for assembling, evaluating, summarizing the evidence, and a multidisciplinary guideline development panel that is free from conflicts of interest.

Specifically, the IGEST addresses 1) the CPGs development process and their reporting, without analysing the content of the recommendations, and 2) the extent to which any bias potentially affecting the recommendations (e.g., literature search, selection, assessment, consistency between evidence and recommendations, multidisciplinary process, and conflict of interest management) is minimised during the development process.

#### Generation of IGEST items (step 2)

To identify appropriate items that fit the IGEST scope, we adopted a deductive approach based on a literature review and assessment of existing tools, followed by an inductive method through focus groups. The combination of both deductive and inductive methods is considered best practice, as a literature review provides the theoretical basis for defining the domain, while the use of qualitative techniques moves the domain from an abstract point to the identification of its manifest forms [21].

##### Literature review (step 2a, Fig. 1)

The results of a systematic review [25] on the appraisal tools for CPGs represented a starting point to identify tools for generating the IGEST items. Additional studies and tools were identified by examining the review reference lists and from its update. Considering the large number of tools identified, only tools subject to any sort of validation studies were considered [1, 15, 16, 26–36]. In addition, international standards for guideline development, such as those of the Institute of Medicine [17, 23], the Guidelines International Network (G-I-N) [3, 17], and the NICE manual [37], were carefully examined. None of the existing instruments fully met the intended scope of the IGEST. However, as among the existing tools the domain related to validity [23] (e.g., the relationship between the evidence and recommendations, the substance and quality of the scientific and clinical evidence cited, and the means used to evaluate the evidence) was almost always present, the ISS project steering group organised the material from the retrieved tools to identify critical elements useful for the IGEST development. The relevant findings were discussed to identify ambiguous, irrelevant, duplicated, or missing items, and a set of potential items that better reflected the scope was selected to generate the initial IGEST items.

##### Focus groups for IGEST draft’s refinement (step 2b, Fig. 1)

To refine the initial version of the IGEST, the inductive method of a focus group was used [38]. Thus, a panel of seven experts, selected on the basis of their relevant experience in CPG development with different academic and professional background and geographical origin, was identified and invited by phone by the ISS project steering group (additional file 1). The experts were asked to declare their financial and intellectual interests by using the ISS form for the declaration of interests; these interests were then assessed and managed according to the ISS policy for the disclosure and management of conflict of interests (https://snlg.iss.it/wp-content/uploads/2021/08/MM_v1.3.2_apr_2019.pdf). The initial IGEST version with all instructions was sent them by mail. Afterwards, a focus group aimed at exploring the experts’ opinions, and thoughts on the relevance and congruence of the selected items, the structure and wording of the items, and the type of expected answers to the questions were also discussed. The raised key points were recorded in field notes taken during the session and later collated by the ISS project steering group, and the IGEST vers.1 was then finalised.

#### Pilot testing (step 3)

To test the feasibility and usability of the tool while identifying its potential practical problems and to establish the approximate time required to complete it, a pilot testing on a sample of seven CPGs was performed by two external researchers (GC, SG) (step 3, Fig. 1).

The piloting CPGs were identified through a rapid search via PubMed using the term: "Practice Guideline" as publication type, then 7 out of the 5498 retrieved records were purposely selected to include different characteristics (e.g., specialties, associations/organizations, target population, countries). Guidelines were considered eligible if they were published after 2016 in English language, and they met the guideline definition proposed by the IOM [17]. We excluded consensus conference, position statement, and any secondary publication of the guidelines.

The researchers were asked to assess the CPGs with the IGEST vers.1 and give suggestions for the IGEST’s clarity and comprehensiveness. The subsequent IGEST vers.2 was tested for content validity.

### Phase 2 - Content validity

#### Content validity index (step 4)

The content validity aims at assessing whether the tool is representative of all aspects of the construct and was established using a rigorous quantification process [19, 20, 39, 40], based on independent consensual judgments (step 4, Fig. 1). The CVI was computed on two levels: item-level CVI (I-CVI) and scale CVI (S-CVI).

##### Content validity index first round (step 4a, Fig. 1)

As suggested by Polit and Beck [20, 39], the ISS steering group invited 14 already known experts with extensive experience in research methodology and CPG development and evaluation with different academic and professional background and geographical origin (additional file 1). The ISS policy for the disclosure and management of conflict of interests was also applied at this step.

It is worth mentioning that the seven experts who participated in the focus group (tool development) were not the same as the 14 experts who participated in the content validation process.

Then, IGEST vers.2 was emailed along with clear and concise instructions on how to rate each item and a spreadsheet to report judgments on the relevance of the IGEST using a four-point Likert scale (1 = not relevant, 2 = somewhat relevant, 3 = quite relevant, 4 = highly relevant), and comments and/or suggestions were requested. All the experts were ensured confidentiality throughout the survey process.

To determine the I-CVI, the number of items rated as ‘relevant’ (rating 3 or 4) was divided by the number of experts. Similarly, S-CVI was calculated using the number of items rated or judged as ‘relevant’ by the experts. An I-CVI score of 0.78 and S-CVI of 0.90 were the minimum acceptable indices [39].

##### Content validity index: second round (step 4b, Fig. 1)

The items that scored a CVI ≥0.78 were retained, while the others were modified or excluded based on the comments provided by the evaluators (step 5, Fig. 1). The experts' comments were appraised by the ISS project steering group, and any differences were resolved by consensus. As a result, the tool was reformulated, and the IGEST vers.3 was sent to the experts or the second round of CVI evaluation. Each expert was asked to express the level of agreement with the amendments made, following the same procedure used in the first CVI evaluation process. At the end of the second round of CVI, the ISS project steering group discussed the results and potential solutions and refined the list of items accordingly.

## Results

### Phase 1 - Tool development

The development and preliminary validation of the IGEST were carried out systematically by following several steps narratively described below and summarised in Table 1.

**Table 1:** IGEST development process by methods and results

	**METHODS**				**RESULTS**	
	**Steps and nature of activity**	**IGEST version**	**Methods**	**People involved**	**Number of items**	**Other modifications**
Phase I Development	1 Define the IGEST scope		Analyse CPGs’ methodological quality standards	ISS project steering group		
	2 Generation of items	IGEST initial version	2a. Literature review and item extraction	ISS project steering group	151 items from literature review	
					32 items after extraction	Removed 119 items for overlapping (*n*=62), other contents (*n*=52), lack of clarity (*n*=5)
		IGEST vers.1	2b. Focus group on IGEST refinement	7 experts	16	Removed and merged 13 and 3 items, respectively. 16 items grouped into 4 dimensions (management of conflict of interest, quality of evidence and consistency, panel composition, and reporting)
	3 Testing feasibility/usability	IGEST vers.2	Pilot testing	2 external researchers	16	Changes in item sequence. Dimension 4 on reporting was considered as primary conditions: - 3 dimensions (management of conflict of interest, quality of evidence and consistency, panel composition)
Phase II Content validity	4 Content validity index	IGEST vers.3	4a. CVI - first round	14 experts	16	Rewording of items Sentence specifications with some footnotes
		IGEST final version	4b. CVI - second round			

#### Generation of IGEST items

##### Literature review

Out of 151 items derived from the retrieved tools (step 2a), 119 were excluded for one or more of the following reasons: overlapping (*n*=62), other contents (*n*=52), lack of clarity (*n*=5). This resulted in the IGEST initial version composed of 32 items to be examined for further refinements.

##### Focus group for IGEST draft’s refinement

To keep the IGEST practical and short, a further 16 items were merged or removed because of redundancy. The remaining 16 items were then grouped into four dimensions: management of conflict of interest, quality of evidence and consistency, panel composition, and reporting. This process culminated in the IGEST vers.1. Regarding the IGEST answer options, the focus group’s experts felt that the likelihood of obtaining more accurate and precise responses would be enhanced by breaking down tool items into more specific criteria related to the CPGs’ quality. Therefore, the choices for the final item pool consisted of four different in-depth criteria scored on a four-point Likert scale (poor, fair, good, excellent).

#### Pilot test

As a result of the pilot testing, five out of the seven (71%) guidelines fulfilled the preliminary conditions and were then fully evaluated. Details of the IGEST evaluation of the seven selected guidelines are reported in Additional file 2.

The included CPGs were published between 2016 and 2019 in different countries (Europe, America, Canada) and covered different medical specialties (neurology, paediatric, public health, oncology, surgery, and dermatology); they varied in length (from 11 to 363 pages) and number of recommendations developed (from 2 to 200); most CPGs developed an own methodology based on international standards for guideline development (i.e., GRADE, SIGN).

The pilot test concluded that the average time required to complete the form was 15 minutes and that the format of the questionnaire was acceptable. Of particular importance, the results of piloting led to some modifications in the item sequence. The two external researchers felt that the four reporting items had to be considered preliminary conditions as they represent the prerequisites to decide on the opportunity to evaluate the other aspects in detail. Following this suggestion, the criteria that address the reporting of the guideline were considered preliminary conditions binary scored (yes/no). At the end of the pilot testing, the IGEST vers.2 was released.

### Phase 2 – Content validity

#### Content validity index

##### First round

During the CVI first round (I-CVI ranged from 0.64 to 1.0, S-CVI = 0.90), the 14 experts suggested that within dimension 2, ‘criterion to rank the quality’ and ‘besides the study type’ were too vague. Thus, to avoid ambiguity, the ISS steering group decided to replace them with ‘study type’ and ‘risk of bias’, respectively. In addition, a better explanation of what ‘GRADE or GRADE-like method’ means was added, and the phrase ‘link between quality of evidence and strength of recommendations’ within all the criteria was added. With regard to dimension 3, the main suggestion was a change of the wording of some criteria to keep a more flexible approach. These refinements ended in the release of the IGEST vers.3. Additional file 3 shows in detail the IGEST vers.3 and the CVI first-round results.

##### Second round

During the CVI second round, a better satisfactory level of I-CVI (from 0.78 to 1.00) and S-CVI (0.90) was achieved (Table 2). The amount of feedback was substantially smaller than in the first round; nevertheless, the experts’ comments resulted in some explanations. Specifically, what is intended with ‘non-financial conflict of interest’, ‘relevant conflict’, and ‘GRADE-like method’ was added as footnotes. After this revision, the final version of the IGEST was drawn. No further change in the IGEST was performed after the revision.

**Table 2:** IGEST final version and CVI second-round score

	No. of agreement	I-CVI
**Preliminary condition**		
a. The full disclosure of any financial conflict of interest (COI) for each decision voted by panellists is reported.	13	0.92
b. The strategy for systematic review of the literature (i.e., search strategy and study selection) is clearly described.	14	1.00
c. A full description of the affiliation and professional profile of panellists is reported.	14	1.00
d. The external review carried out by independent experts is reported.	12	0.85
**Dimension 1. Conflict of interest**		
1. The guideline should describe how any identified conflicts were recorded and resolved.	12	0.85
2. Non-financial COI is managed.	11	0.78
3. COI of any guideline development group members are examined and managed by an oversight committee.	12	0.85
4. Chair and co-chair are not allowed to have any relevant^2^ financial COI.	11	0.78
**Dimension 2. Quality and consistency**		
5. Quality of evidence is rated according to study type, and there is no explicit link between quality of evidence and strength of recommendations.	14	1.00
6. Quality of evidence is rated according to study type, and there is an explicit link between the quality of evidence and strength of recommendations.	13	0.92
7. Quality of evidence is rated according to both study type and risk of bias, and there is an explicit link between the quality of evidence and strength of recommendations.	11	0.78
8. Rating quality of evidence and grading strength of recommendations are based on GRADE or GRADE-like method.	14	1.00
**Dimension 3. Panel composition**		
9. Only one clinical specialty is involved.	12	0.85
10. More than one clinical specialty is involved.	12	0.85
11. Different relevant clinical specialities, general practitioners, and other professional groups are involved.	13	0.92
12. Different relevant clinical specialities, general practitioners, other professional groups, and at least one patient representative are involved.	14	1.00

### The IGEST tool

The final version of the IGEST includes four preliminary conditions and 12 criteria organised into three dimensions: (i) the management of conflict of interest, where a positive assessment means disclosing and managing interests in the perspective of making decisions on the member’s participation in the formulation of final recommendations; (ii) the quality of evidence and the coherence between evidence and recommendations, which are achievable through a rigorous systematic review with appropriate evidence rating and a transparent approach when moving from critical appraisal of evidence and formulation of recommendations; (iii) the panel composition with a broad representation of expertise from several disciplines and professional fields.

The IGEST is intended to be used by groups interested in adopting/adapting existing CPGs to their own context to facilitate the process of quickly assessing their quality and decide whether to use them as source CGPs. The IGEST should enable us to eliminate CPGs that are clearly not trustworthy while retaining those worth of a more detailed assessment with extensive instruments. It means that the IGEST is a screening tool, and its use is considered as preliminary and complementary rather than a substitute for more complex instruments (e.g., AGREE). Then, the guidelines that pass the IGEST screening will be assessed with reference to aspects mainly related to recommendations applicability/ transferability, as foreseen by GRADE-ADOPOLMENT [10].

The IGEST is a generic tool that can be applied to guidelines of any specialties, target population, and health care setting. It is recommended that the appraisers are familiar with the guideline development process and critical appraisal, nevertheless the level of experience in the evaluators was not part of our investigation. Moreover, although we did not test the number of appraisers needed for the most reliable assessment, we recommend that at least two appraisers should assess each guideline independently because it is highly expected that two appraisers will enhance reliability through a discussion of any discrepancy and reaching consensus.

Our pilot testing experience has demonstrated that rating a guideline with the IGEST required about 15 minutes per appraiser, however additional time must be spent to identify any supporting documents and resources (e.g., appendices, supplements) and all other relevant information related to the CPGs as well as to resolve discrepancies between appraisers.

The IGEST scoring system includes only a yes/no answer options for the preliminary conditions, and a four-point Likert scale (poor, fair, good, excellent) for the three dimensions. As there was *a priori* reason for some criteria to have greater weight than others, it was decided that the Likert points (poor, fair, good, excellent) have different meanings according to the dimension they refer to. For the guideline to be considered trustworthy, the preliminary conditions need to be fulfilled to proceed with the screening criteria, while the minimum score assigned to each dimension needs to be ‘fair’. As the IGEST was built with some decision points (yes/no, minimum score), when using the IGEST it is recommended to draw a sort of flow chart that visually displays the assessment sequence. The IGEST tool is completed in two main steps: (1) assessment of the preliminary conditions mainly focused on reporting elements and (2) identification of concerns about aspects regarding guidelines’ trustworthiness.

When interpreting the results, several scenarios could arise during the screening process, some examples are presented in Additional file 2 and summarised below.

During the step for selecting CPGs for further evaluation, in the case where the CPG has not fulfilled all the preliminary conditions, it must be rejected with no further assessment. Reasons for the decision should be discussed and recorded.

On the contrary, in the case where the preliminary conditions are all fulfilled, the appraiser can move to the second step aimed at rating the three IGEST dimensions. All three dimensions must be rated at least as “fair” for the guideline to be considered trustworthy. For example, if the appraisers rated dimension 1 (management of COI) as “poor”, while the others two (quality of evidence and panel composition) are “excellent”, the CGP will not be recommended for adoption/adaptation. Conversely, if all the three dimensions are rated as “fair”, the CPGs will be recommended. The evaluation should be transparent, and for each CGP the decision to include or exclude should be recorded, along with the reasons for any exclusion.

The final version of the questionnaire comprising 16 items and its scoring system are presented in Additional file 4.

## Discussion

We described the development and validation process of the IGEST, a new instrument to quickly evaluate and screen the quality of international CPGs for adoption/adaptation to the local context. We developed the IGEST through a comprehensive development process, from the use of a systematic review to content validity evaluation from experts’ point of view. Interestingly, during the IGEST initial item generation, several criteria were found to be common to most of the retrieved instruments, causing significant overlapping. However, even if the IGEST final version includes some aspects already present in other tools, it differs from other checklists in scope and format, mainly because it intends to support adoption or adaptation of international CPGs to the local context, as endorsed by GRADE-ADOPOLMENT [10].

It is worth noting that dimension 1 (management of conflict of interest) was felt by the panel to be less relevant than the others, even though the IOM recommendations emphasise its importance [23, 24]. Since few tools cover that dimension [25, 41], it is reasonable to think that the panel of experts was less aware of how inappropriate conflict management negatively affects guideline quality. Indeed, during the IGEST development phase, the ISS project steering group paid great attention to combine the most used validity elements (e.g., literature search and selection, the use of the evidence to generate recommendations) with those less used (e.g., conflict of interest, patient–caregiver involvement, panel composition) [25, 42]. This was because CPGs are more than a systematic review of relevant evidence; rather, they are a group process where the recommendations are influenced by identification, interpretation, and judgments by the guideline development group. This combination of elements represents the ISS project steering group’s attempt to mitigate the increasing confusion of CPGs with evidence summaries [25, 43].

With respect to the IGEST scoring system, it is important to highlight that conversely to other widely used tools [15, 26], the IGEST provides thresholds to classify CPGs as ‘acceptable’ or ‘unacceptable’, mainly to support users in decision making.

With regards to IGEST application considerations, it is important to highlight that the IGEST is meant to allow users to have a quick understanding of the rigor of the method used during a CPG development process and thus, before embarking on an extensive evaluation for its adoption/adaptation. For instance, during the GRADE ADOLOPMENT steps for adoption, adaptation and de novo development [10] or the ADAPTE process for guideline adaptation [12], the IGEST can be used for quickly identifying source guidelines, as it provides information about the quality of the retrieved guidelines (i.e., currency, consistency) capable to support users in decision making around adaptation.

Furthermore, it is important to underline that, as some of the IGEST items are common to other tools, once the screening with the IGEST is completed and the decision to evaluate CPGs in detail with extensive instruments is made, the aspects already evaluated with the IGEST should not be re-evaluated, thus avoiding duplication of efforts. Reasonably, we assumed that after the IGEST assessment, the following extensive evaluation requires considerably less time and personnel resources, and those CPGs that fulfil the IGEST standards will increase their likelihood of achieving high scores when evaluated with extensive assessment tools (i.e., AGREE II, GLIA).

### Strengths and limitations

We acknowledge several limitations in the process of developing the IGEST. As we did not perform a new systematic review to find existing instruments, we may have missed important instruments. To minimise this possibility, we examined many standards and manuals on the guideline development process.

Although we outlined that the IGEST can be completed relatively quickly, we did not test whether it requires specific training before being used and the exact number of appraisers needed for the most reliable assessment. In the future, it would be useful to investigate to what extent the level of experience and the number of appraisers would increase the reliability of the assessments. Moreover, it is important to recognize that all the participants in the focus group and validation process were affiliated with Italian agencies/organizations, so it would be necessary to involve a wider international group to ensure the IGEST considers all important aspects.

In the present paper we explored the validity of the IGEST, while its psychometric properties and reliability will be detailed in a further study where it will be applied to a wider selection of CGPs.

During the IGEST development process, the ISS steering group provided a focused discussion regarding the IGEST scope and its initial development, and then several groups were involved in the process. Despite these efforts, we feel that a wider representation would have benefited the IGEST development, so a website section will be dedicated to collect feedback from a wider group of stakeholders, while the ISS group will use the IGEST to screen the international guidelines to be posted on the SNLG website (https://snlg.iss.it). Moreover, we are currently developing an electronic version of IGEST that will provide a more efficient way to visualize and report results.

## Conclusion

The IGEST is a promising tool that can be used to screen CPGs and inform guideline developers about the option to adopt/adapt those guidelines considered trustworthy. It offers a standardised approach to give insight into the degree of international CPG trustworthiness based on the most widely accepted standards for the guideline development process. The possibility to publicly post on the SNLG website the international CPGs that met stringent inclusion criteria can assist developers in guideline adoption/adaptation for efficient resource utilisation while saving time and resources. It might increase the likelihood that international CPGs will be adopted/adapted to the local context instead of developing de novo guidelines, since it allows a quick screening of existing guidelines trustworthiness and provides an acceptability threshold that supports the decision-making processes. Moreover, it might contribute towards sustained improvement of international collaboration in guideline development and implementation. As a new screening tool, the IGEST will require regular testing and revision to refine and weigh its items, it will be possible thanks to stakeholders’ comments and a constructive discussion with a wide range of guideline developers.

## Supplementary Information

**Additional file 1.**

**Additional file 2.**

**Additional file 3.**

**Additional file 4.**

## Data Availability

The datasets used and/or analyzed during the current study are available from the corresponding author on reasonable request.

## Abbreviations

IGEST: International Guideline Evaluation Screening Tool
CPGs: Clinical Practice Guidelines
ISS: Istituto Superiore di Sanità (Italian National Institute of Health)
SNLG: Sistema Nazionale Linee Guida (Italian National Guidelines System)
CVI: Content validity index
I-CVI: Item-level content validity index
S-CVI: Scale content validity index
