# Supplementary material for: The International Guideline Evaluation Screening Tool (IGEST): development and validation
Source: BMC Med Res Methodol. 2022 May 10;22:134. doi: 10.1186/s12874-022-01618-5 (PMC9088113; doi:10.1186/s12874-022-01618-5)
Supplement: Supplementary file 1 — Additional file 1. [file 12874_2022_1618_MOESM1_ESM.docx]

**Additional file 1**. Experts’ characteristics

| Focus group participants’ characteristics (N=7) | | | | |  |
| --- | --- | --- | --- | --- | --- |
|  | **Academic/professional background** | **Gender** | **Geographical origin** | **Years of experience in CPGs development** | **Conflict of interests** |
| 1 | MDMPH, epidemiologist | F | South Italy | 10 | None |
| 2 | MDMPH, public health specialist | M | South Italy | 8 | None |
| 3 | MA, PhD, anthropologist and public health specialist | F | South Italy | 6 | None |
| 4 | PhD, Nurse, research methodologist | F | Middle Italy | 8 | None |
| 5 | PhD, Nurse, research methodologist | F | Middle Italy | 5 | None |
| 6 | PhD, Nurse, research methodologist | M | North Italy | 7 | None |
| 7 | MSPhty, PhD, research methodologist | F | North Italy | 8 | None |

| Characteristics of the experts who participated in the content validation process (N=14) | | | | |  |
| --- | --- | --- | --- | --- | --- |
|  | **Academic/professional background** | **Current position and affiliation** | **Gender** | **Years of experience in CPGs development** | **Conflict of interests** |
| 1 | MD, Internal Medicine | Internal Medicine Unit, ASL4 Chiavarese | M | 10 | None |
| 2 | MD, Neurologist, methodologist | IRCCS Istituto Scienze Neurologiche di Bologna | M | 13 | None |
| 3 | MD, epidemiologist | Head of the Scientific Research Unit, Dept. of Epidemiology, ASL Roma1 Regione Lazio. Member of the Italian GRADE Centre. | F | 25+ | None |
| 4 | Statistician, PhD | Head, Unit of systematic reviews and guidelines production Istituto di Ricerche farmacologiche Mario Negri IRCCS. Member of the Italian GRADE Centre | F | 15 | None |
| 5 | RN, PhD | Associate Professor | M | 25+ | None |
| 6 | RN, PhD | Associate Professor | F | 15 | None |
| 7 | MD, MS, epidemiologist | Consultant at Department of Epidemiology, Lazio Regional Health Service, Rome, Italy. Member of the Italian GRADE Center | F | 15 | None |
| 8 | RN, PhD student | Head of Health Professions Research and Development Unit, IRCCS Policlinico San Donato | M | 3 | None |
| 9 | MD, Neurologist, epidemiologist | Director of WHO Collaborating Centre in Evidence-Based Research Synthesis and Guideline Development, Bologna | M | 20 | None |
| 10 | MD, clinical epidemiologist | Head, assessment of clinical appropriateness and effectiveness at Modena University Hospital | F | 12 | None |
| 11 | MD, MIHMEP, PhD in Clinical and Experimental Medicine | Head of Unit, "S.S. Marketing e Libera Professione" at University Hospital "Azienda Ospedaliero-Universitaria di Novara | M | 15 | None |
| 12 | MD, internal medicine, oncology | Director, Medical Oncology, IRCCS Ospedale Sacro Cuore Don Calabria, Negrar, VR | F | 25 | None |
| 13 | MSc, PhD, Methodologist | Project Officer at European Commission- Joint Research Centre,  Ispra | M | 10 | None |
| 14 | MSPhty, PhD, methodologist | Researcher at the Unit of Clinical Epidemiology, IRCCS Istituto Ortopedico Galeazzi | F | 3 | None |
