# Supplementary material for: The International Guideline Evaluation Screening Tool (IGEST): development and validation
Source: BMC Med Res Methodol. 2022 May 10;22:134. doi: 10.1186/s12874-022-01618-5 (PMC9088113; doi:10.1186/s12874-022-01618-5)
Supplement: Supplementary file 2 — Additional file 2. [file 12874_2022_1618_MOESM2_ESM.docx]

| **ADDITIONAL FILE 2.**  Description of piloting guideline evaluations | | | | | | | | | |
| --- | --- | --- | --- | --- | --- | --- | --- | --- | --- |
| **Guideline reference** | **Preliminary conditions** | | | | | **Dimension 1** | **Dimension 2** | **Dimension 3** |  |
|  | Financial COI disclosure | Systematic review strategy | Panelists’  profile | External review | *Final Evaluation* | Management of COI | Quality of evidence | Panel composition | *Recommended adaptation* |
| Belyaev I, Dean A, Eger H, Hubmann G, Jandrisovits R, Kern M, Kundi M, Moshammer H, Lercher P, Müller K et al: EUROPAEM EMF Guideline 2016 for the prevention, diagnosis and treatment of EMF-related health problems and illnesses. Reviews on environmental health 2016, 31(3):363-397. | Y | N | N | N | Rejected |  | | | |
| Warner JJ, Harrington RA, Sacco RL, Elkind MSV: Guidelines for the Early Management of Patients With Acute Ischemic Stroke: 2019 Update to the 2018 Guidelines for the Early Management of Acute Ischemic Stroke. Stroke 2019, 50(12):3331-3332. | Y | Y | Y | Y | Accepted | G | G | F | Yes |
| Fowler KJ, Kaur H, Cash BD, Feig BW, Gage KL, Garcia EM, Hara AK, Herman JM, Kim DH, Lambert DL et al: ACR Appropriateness Criteria(®) Pretreatment Staging of Colorectal Cancer. Journal of the American College of Radiology : JACR 2017, 14(5s):S234-s244. | Y | Y | Y | N | Rejected |  | | | |
| Smith CH, Jabbar-Lopez ZK, Yiu ZZ, Bale T, Burden AD, Coates LC, Cruickshank M, Hadoke T, MacMahon E, Murphy R et al: British Association of Dermatologists guidelines for biologic therapy for psoriasis 2017. The British journal of dermatology 2017, 177(3):628-636. | Y | Y | Y | Y | Accepted | G | E | F | Yes |
| Puligandla PS, Skarsgard ED, Offringa M, Adatia I, Baird R, Bailey M, Brindle M, Chiu P, Cogswell A, Dakshinamurti S et al: Diagnosis and management of congenital diaphragmatic hernia: a clinical practice guideline. CMAJ : Canadian Medical Association journal = journal de l'Association medicale canadienne 2018, 190(4):E103-e112. | Y | Y | Y | Y | Accepted | G | G | G | Yes |
| Tremblay MS, Chaput JP, Adamo KB, Aubert S, Barnes JD, Choquette L, Duggan M, Faulkner G, Goldfield GS, Gray CE et al: Canadian 24-Hour Movement Guidelines for the Early Years (0-4 years): An Integration of Physical Activity, Sedentary Behaviour, and Sleep. BMC public health 2017, 17(Suppl 5):874. | Y | Y | Y | Y | Accepted | P | E | E | No |
| Yancy CW, Jessup M, Bozkurt B, Butler J, Casey DE, Jr., Colvin MM, Drazner MH, Filippatos GS, Fonarow GC, Givertz MM et al: 2017 ACC/AHA/HFSA Focused Update of the 2013 ACCF/AHA Guideline for the Management of Heart Failure: A Report of the American College of Cardiology/American Heart Association Task Force on Clinical Practice Guidelines and the Heart Failure Society of America. Journal of cardiac failure 2017, 23(8):628-651. | Y | Y | Y | Y | Accepted | E | G | F | Yes |

Legend: Y (yes) N (no)

P (poor) F (fair) G (good) E (excellent)

| Clinical practice guidelines characteristics | | | | | | |
| --- | --- | --- | --- | --- | --- | --- |
| Guideline title | Publication year | Region | Specialty area | Method | Topic | N° of recommendations |
| EUROPAEM EMF Guideline 2016 for the prevention, diagnosis and treatment of EMF-related health problems and illnesses | 2016 | Europe | Public health | Not specified | EMF (electromagnetic field) exposure and related health risks | 7 |
| Guidelines for the Early Management of Patients with Acute Ischemic Stroke | 2019 | United States of America | Neurology | American College of Cardiology/  American Heart Association 2015 Class of Recommendations and Levels of Evidence and the new American Heart  Association guidelines format | Early Management of Patients  With Acute Ischemic Stroke | 170 |
| ACR Appropriateness Criteria (®) Pretreatment Staging of Colorectal Cancer | 2017 | United States of America | Oncology | RAND/UCLA Appropriateness  Method and Grading of Recommendations Assessment, Development, and Evaluation or GRADE | Gastrointestinal Imaging | 2 |
| British Association of Dermatologists guidelines for biologic therapy for psoriasis | 2017 | UK | Dermatology | Grading of Recommendations Assessment, Development  and Evaluation (GRADE) | Treatment of psoriasis | 57 |
| Diagnosis and management of congenital diaphragmatic hernia: a clinical practice guideline | 2018 | Canada | Surgery | Taxonomy scheme for grading of evidence.  Modified Delphi consensus framework | Congenital  diaphragmatic hernia | 22 |
| Canadian 24-Hour Movement Guidelines for the Early Years (0-4 years): An Integration of Physical Activity, Sedentary Behaviour, and Sleep | 2017 | Canada | Public health | Grading of  Recommendations Assessment, Development, and Evaluation (GRADE) | The balance of movement behaviours across the whole day for children (0-4) | 8 |
| ACCF/AHA Guideline for the Management of Heart Failure: A Report of the American College of Cardiology/American Heart Association Task Force on Clinical Practice Guidelines and the Heart Failure Society of America | 2017 | United States of America | Cardiology | The Class of Recommendation (COR) indicates the strength of the recommendation, encompassing the estimated magnitude and certainty of benefit in proportion to risk. The Level of Evidence (LOE) rates the quality of scientific evidence | Management of Heart Failure | 28 |
