# Supplementary material for: The International Guideline Evaluation Screening Tool (IGEST): development and validation
Source: BMC Med Res Methodol. 2022 May 10;22:134. doi: 10.1186/s12874-022-01618-5 (PMC9088113; doi:10.1186/s12874-022-01618-5)
Supplement: Supplementary file 3 — Additional file 3. [file 12874_2022_1618_MOESM3_ESM.docx]

| **Additional file 3.** IGEST ver.3 and I-CVI score | | |
| --- | --- | --- |
|  | N. of agreement | I-CVI |
| **Preliminary condition** |  |  |
| The full disclosure of any financial COI for each decision voted by panellists is reported | 13 | 0.92 |
| The strategy for systematic review of literature (i.e.: search strategy and study selection) is clearly described | 14 | 1.00 |
| A full description of affiliation and professional profile of panellists is reported. | 14 | 1.00 |
| External review carried out by independent experts is reported | 12 | 0.85 |
| **Dimension 1 Conflict of interest** |  |  |
| The guideline should describe how any identified conflicts were recorded and resolved | 12 | 0.85 |
| Non-financial conflict of interest (COI) is managed | 11 | 0.78 |
| COI of any guideline development group members are examined and managed by an oversight committee | 12 | 0.85 |
| Chair and co-chair are not allowed to have any relevant^2^ financial COI | 11 | 0.78 |
| **Dimension 2 Quality and consistency** |  |  |
| Only one criterion for rating quality of evidence is adopted | 9 | 0.64 |
| At least one criterion for rating quality (level) of evidence and clear criteria linking quality (level) of evidence to strength of recommendations are adopted | 10 | 0.71 |
| At least one more criterion for rating the quality (level) of evidence besides the study type and clear criteria linking quality (level) of evidence to strength of recommendations are adopted | 11 | 0.78 |
| GRADE or GRADE-like method is adopted | 9 | 0.64 |
| **Dimension 3 Panel composition** |  |  |
| Only one clinical specialty is involved | 12 | 0.85 |
| Some, but not all, related specialties and no general practitioner are involved | 10 | 0.71 |
| All relevant specialties, general practitioner and health care providers are involved, and no patient representative are included | 9 | 0.64 |
| All relevant clinical specialities, general practitioners, and health care providers and at least one patient representative voting or consulted before the release of recommendation is involved | 14 | 1.00 |
