# Supplementary material for: The International Guideline Evaluation Screening Tool (IGEST): development and validation
Source: BMC Med Res Methodol. 2022 May 10;22:134. doi: 10.1186/s12874-022-01618-5 (PMC9088113; doi:10.1186/s12874-022-01618-5)
Supplement: Supplementary file 4 — Additional file 4. [file 12874_2022_1618_MOESM4_ESM.docx]

ADDITIONAL FILE 4

**International Guidelines Evaluation Screening Tool (IGEST)**

The criteria hereby presented are adopted to ascertain whether to include international Clinical Practice Guidelines (CPGs) in the international CPGs database held by the SNLG-ISS.

Among the 16 criteria, 12 are organized in 3 dimensions and 4 are set as preliminary conditions. The tool is designed to screen CPGs towards the above-mentioned criteria. Nevertheless, it is recommended that users assess thoroughly the CPGs quality by adopting the tools suggested in the specific sections of the SNLG-ISS web site (i.e., AGREE instruments).

**CPGs are included in the SNLG-ISS if:**

- **all preliminary conditions are fulfilled AND**
- **dimensions 1-3 are rated at least as fair**

# Preliminary conditions

1. The full disclosure of any financial conflict of interest (COI) for each decision voted by panellists is reported.
2. The strategy for systematic review of the literature (i.e., search strategy and study selection) is clearly described.
3. A full description of the affiliation and professional profile of panellists is reported.
4. The external review carried out by independent experts is reported.

*If all preliminary conditions are fulfilled, you can proceed; otherwise reject.*

# Dimension 1: management of conflict of interest (COI)

Criteria:

- 1. The guideline should describe how any identified conflicts were recorded and resolved.
  2. Non-financial COI ^[[1]](#footnote-1)^are managed.
  3. COI of any guideline development group members are examined and managed by an oversight committee.
  4. Chair and co-chair are not allowed to have any relevant^[[2]](#footnote-2)^ financial COI.

Rating scale:

- **Poor**: no criteria met.
- **Fair**: only criterion 1 met.
- **Good**: criterion 1 + any of the remaining 2-4 met.
- **Excellent:** all the criteria met.

# Dimension 2: rating quality of evidence and link with the strength of recommendations

Criteria:

- 1. Quality of evidence is rated according to study type and there is no explicit link between quality of evidence and strength of recommendations.
  2. Quality of evidence is rated according to study type and there is an explicit link between the quality of evidence and strength of recommendations.
  3. Quality of evidence is rated according to both study type and risk of bias and there is

an explicit link between the quality of evidence and strength of recommendations.

- 1. Rating quality of evidence and grading strength of recommendations are based on GRADE or GRADE-like method^[[3]](#footnote-3)^.

Rating scale:

- - **Poor**: criterion 5 met.
  - **Fair**: criterion 6 met.
  - **Good**: criterion 7 met.
  - **Excellent**: criterion 8 met.

# Dimension 3: panel composition

Criteria:

- 1. Only one clinical specialty is involved.
  2. More than one clinical specialty is involved.
  3. Different relevant clinical specialities, general practitioners, and other professional groups are involved.
  4. Different relevant clinical specialities, general practitioners, other professional groups and

at least one patient representative are involved.

Rating scale:

- - **Poor**: only criterion 9 met.
  - **Fair**: criterion 10 met.
  - **Good**: criterion 11 met.
  - **Excellent**: criterion 12 met.

1. Eg: professional or academic advantage. [↑](#footnote-ref-1)
2. A relevant COI exists if it influences the direction or strength of a recommendation (GIN 2015). [↑](#footnote-ref-2)
3. Eg: considering multiple criteria addressing quality of evidence and relationship with strength of recommendations. [↑](#footnote-ref-3)
